# Application effect and teaching evaluation of case-based learning combined with ChatGPT in ophthalmology clinical teaching

**DOI:** 10.3389/fmed.2026.1885204

**Published:** 2026-07-03

**Authors:** Yuan Shen, Yuxiao Chen, Mengyao Li, Xiang Gu, Ai Zhuang

**Affiliations:** 1 Department of Ophthalmology, Shanghai Ninth People’s Hospital, Shanghai Jiao Tong University School of Medicine, Shanghai, China; 2 Shanghai Jiao Tong University School of Medicine, Shanghai, China

**Keywords:** case-based learning, ChatGPT, clinical teaching, educational evaluation, ophthalmology

## Abstract

**Objective:**

To investigate the effectiveness of a case-based learning (CBL) model integrated with ChatGPT in ophthalmology clinical teaching and to compare its educational outcomes with those of the traditional multimedia lecture-based approach.

**Methods:**

A total of 98 fifth-year clinical medicine students from Shanghai Jiao Tong University School of Medicine (Class of 2021) were randomly assigned to an experimental group (CBL + ChatGPT, *n* = 49) or a control group (traditional lecture-based teaching, *n* = 49). The teaching intervention lasted for 8 weeks, with one 90-min session per week. The experimental group adopted a standardized human-AI interaction protocol, including a structured questioning framework, no fewer than three rounds of dialog, and real-time teacher supervision and correction. The control group received conventional multimedia lectures. Outcomes included theoretical knowledge examination scores, case analysis scores, recognition rates of the teaching model across seven dimensions, and overall teaching satisfaction. Subgroup analyses were further conducted according to students’ baseline academic performance (high-, medium-, and low-foundation groups).

**Results:**

The experimental group achieved significantly higher theoretical examination scores than the control group (86.4 ± 5.2 vs. 78.9 ± 6.1, *P* < 0.01), as well as higher case analysis scores (84.7 ± 6.3 vs. 74.2 ± 7.5, *P* < 0.01). Recognition rates across all seven evaluation dimensions, including interactivity, immediate feedback, and clinical relevance, were significantly higher in the experimental group (all *P* < 0.05). The overall satisfaction rate was 89.8% in the experimental group and 63.3% in the control group (*P* < 0.01). Subgroup analysis demonstrated that students with weaker academic foundations benefited most from the intervention, showing the greatest improvement in scores (+12.3 points, *P* < 0.01). These students also reported significantly higher recognition of personalized learning experiences than students with stronger academic backgrounds (95.2% vs. 81.2%, *P* < 0.05).

**Conclusion:**

The CBL teaching model integrated with ChatGPT significantly improves both objective learning outcomes and subjective satisfaction in ophthalmology clinical education, particularly among students with weaker academic foundations. This teaching approach demonstrates substantial potential for broader implementation in medical education.

## 1 Introduction

Artificial intelligence (AI) technologies are increasingly transforming medical education by enabling adaptive, interactive, and learner-centered learning environments (1–4). Large language models represented by ChatGPT have demonstrated promising performance in medical knowledge assessment (5–7), clinical reasoning support (8, 9), improvement in clinical consultation and patient interviewing skills (10, 11), and personalized educational assistance (12, 13), thereby attracting growing attention in healthcare education research. Ophthalmology education remains particularly challenging because of the specialty’s complex anatomical structures (14, 15), strong reliance on visual interpretation (16, 17), and limited curricular exposure during undergraduate medical training (18, 19). Traditional lecture-based teaching methods often emphasize passive knowledge acquisition and may inadequately support the development of clinical reasoning and individualized learning (20–22). Therefore, innovative teaching approaches that enhance interactivity and clinical integration are urgently needed in ophthalmology education.

Case-based learning (CBL) has been widely adopted in medical education because it promotes active participation, contextual learning, and clinical problem-solving abilities (23–25). Recently, several studies have explored the use of ChatGPT in medical teaching activities, including problem-based learning (26, 27), rare disease education (28), and clinical discussion training (29, 30). Existing evidence suggests that AI-assisted teaching may improve learner engagement and self-directed learning efficiency (31). However, current studies still present several important limitations. First, many studies rely primarily on subjective learner feedback or single-group pre-post designs, lacking rigorous randomized controlled comparisons and objective learning outcome assessments (32). Second, the differential educational impact on students with varying academic foundations has not been adequately investigated (33). Third, standardized workflows for human-AI interaction and teacher supervision have been insufficiently described, limiting reproducibility and practical implementation (34). Moreover, evidence regarding ChatGPT-assisted ophthalmology teaching remains limited (35).

Therefore, this study aimed to evaluate the effectiveness of a CBL teaching model integrated with ChatGPT in ophthalmology clinical education using a randomized parallel-controlled design. Both objective learning outcomes and subjective teaching evaluations were assessed. In addition, subgroup analyses according to baseline academic performance were conducted to investigate the adaptability of this teaching model among students with different learning foundations. By establishing a standardized human-AI interaction framework combined with real-time teacher supervision, this study sought to provide reproducible evidence regarding the educational value and practical feasibility of ChatGPT-assisted CBL in ophthalmology teaching.

## 2 Materials and methods

### 2.1 Study participants and group allocation

A total of 98 fifth-year clinical medicine students from Shanghai Jiao Tong University School of Medicine (Class of 2021) were enrolled in this study. Using a random number table managed by an independent administrative staff member, participants were randomly assigned to either the experimental group (CBL + ChatGPT, *n* = 49) or the control group (traditional multimedia lecture-based teaching, *n* = 49). Given that the sample size was constrained by the natural class size, a *post hoc* power analysis was performed, confirming sufficient power to detect significant inter-group differences.

The two groups demonstrated high baseline comparability. The study cohort included 48 males and 50 females aged 22–24 years (mean age: 23.2 ± 0.6 years). No statistically significant differences were observed between the two groups regarding age, sex distribution, or comprehensive medical examination scores (*P* > 0.05).

This study was approved by the Institutional Ethics Committee of Shanghai Ninth People’s Hospital, Shanghai Jiao Tong University School of Medicine. All participants provided written informed consent after receiving detailed study disclosures. They were expressly informed that participation was entirely voluntary and would have no bearing on their course grades or academic evaluations.

### 2.2 Teaching intervention

The teaching intervention lasted 8 weeks, consisting of one 90-min session per week. Eight representative ophthalmic cases were selected, including cataract, glaucoma, retinal detachment, diabetic retinopathy, macular degeneration, keratitis, strabismus, and ocular trauma. Each session centered on one clinical case. All sessions were delivered by the same group of senior ophthalmologists who underwent standardized training before the study.

#### 2.2.1 Experimental group: CBL combined with ChatGPT

**FIGURE 1:**
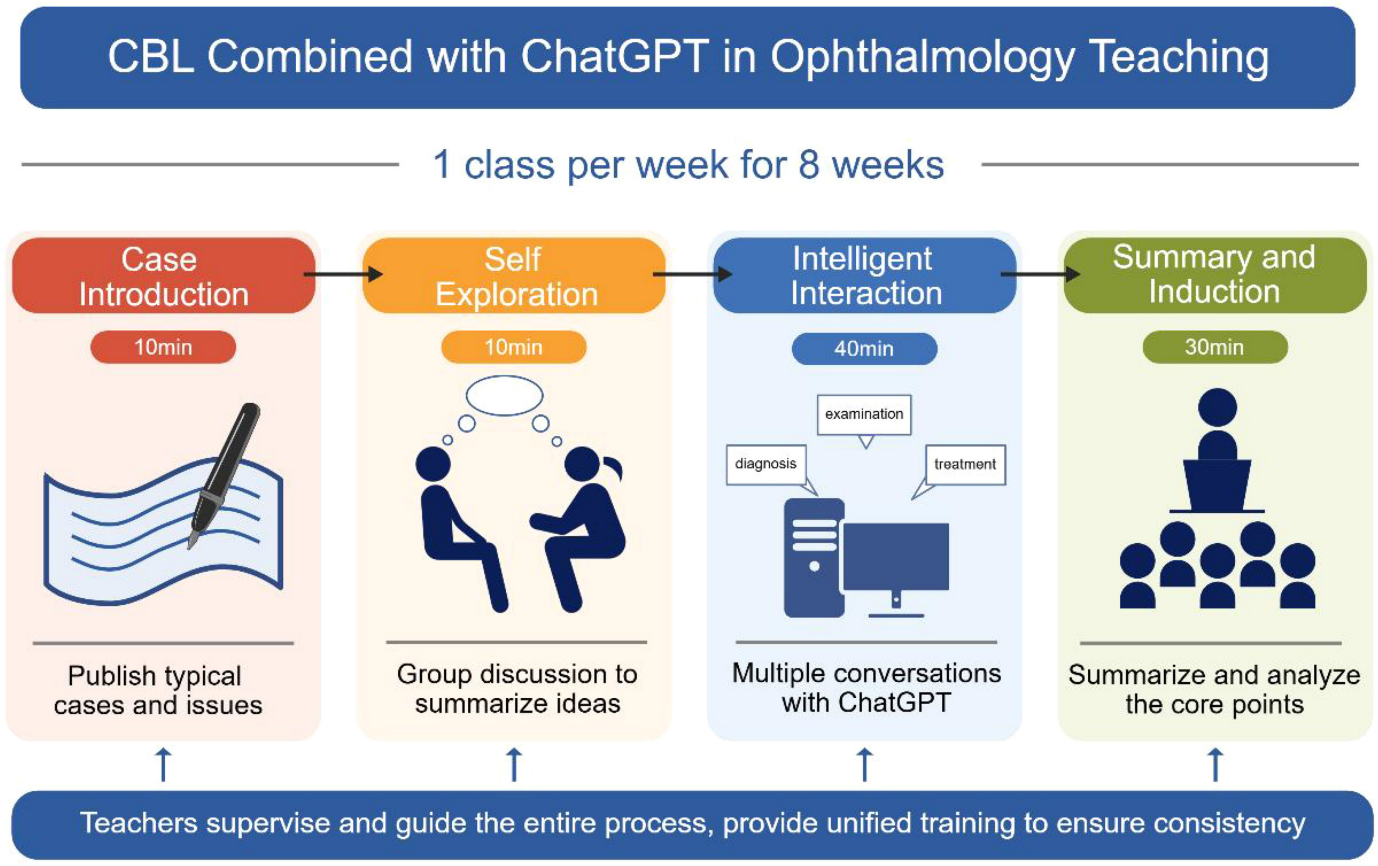
Schematic diagram of CBL combined with ChatGPT teaching process.

The teaching process was conducted according to a standardized workflow (Figure 1).

##### 2.2.1.1 Case introduction

Teachers selected representative ophthalmic cases containing chief complaints, medical histories, and auxiliary examination results according to the curriculum requirements and students’ knowledge levels. A series of progressively structured questions were designed, including preliminary diagnosis, diagnostic basis, differential diagnosis, further examinations, and treatment principles. Cases and guiding questions were released through an online teaching platform to facilitate students’ initial understanding of the core content. This stage lasted 10 min.

##### 2.2.1.2 Group discussion

Students were divided into groups of 8–10 members for open discussion. They were encouraged to summarize diagnostic and therapeutic reasoning using mind maps. ChatGPT was not introduced during this phase, with the aim of strengthening students’ independent analytical ability and teamwork skills based on existing knowledge. This stage lasted 10 min.

##### 2.2.1.3 Human-AI interaction

ChatGPT (OpenAI, GPT-4 architecture accessed in January-March 2026) was used in the teaching process. Teachers designed a structured questioning framework in advance, requiring each student group to complete at least three rounds of interaction with ChatGPT according to a five-step sequence (Table 1).

**TABLE 1:** Standardized human-AI interaction framework and time allocation.

Stage	Prompt template	Suggested time
Clarification of diagnosis	“Please provide a preliminary diagnostic analysis based on the case information and list the diagnostic evidence.”	5 min
Differential diagnosis	“What differential diagnoses should also be considered, and what are the distinguishing features?”	5 min
Additional examinations	“What auxiliary examinations are necessary to confirm the diagnosis, and what is their clinical significance?”	5 min
Treatment decision-making	“What are the treatment principles and specific management plans for this case? Are individualized treatment strategies needed?”	5 min
Prognostic evaluation	“What potential complications may occur, and what are the key factors affecting prognosis?”	5 min

Each group was required to screen, organize, and revise the responses generated by ChatGPT. Teachers supervised the entire process and immediately corrected outdated or guideline-inconsistent information generated by ChatGPT while guiding students to critically evaluate the rationality and limitations of AI-generated content. This phase lasted 25 min.

Simultaneously, each group compared ChatGPT-generated suggestions with their original discussion outcomes formulated before the AI interaction. Differences were documented and analyzed to identify potential causes, including knowledge gaps, deviations in clinical reasoning, or limitations of the AI model. The groups subsequently compiled a final case analysis report. This stage lasted 15 min.

##### 2.2.1.4 Teacher summary and consolidation

Teachers summarized the core knowledge points and standardized diagnostic and therapeutic procedures related to each case. Comparisons between ChatGPT-generated suggestions and actual clinical pathways were analyzed to emphasize the importance of individualized patient factors in clinical decision-making. Teachers also addressed common issues identified across groups and answered students’ questions. This stage lasted 30 min.

#### 2.2.2 Control group: traditional multimedia lecture-based teaching

The control group received instruction using the same clinical cases and the same teaching faculty. Teaching followed a conventional linear format consisting of etiology, pathology, clinical manifestations, diagnosis, and treatment using PowerPoint-based lectures. Each session included 15 min of case analysis and 75 min of systematic lectures. ChatGPT was not introduced, and no group discussions were conducted.

### 2.3 Outcome measures

The evaluation matrix comprised objective learning outcomes and subjective teaching evaluations. Outcome assessors responsible for grading the theoretical examinations and case analysis assessments were blinded to students’ group allocation throughout the study.

#### 2.3.1 Objective learning outcomes

##### 2.3.1.1 Theoretical knowledge examination

Students completed ophthalmology theoretical examinations before and after the intervention (maximum score: 100 points). The examination included 15 single-choice questions (2 points each), four terminology explanation questions (5 points each), and two essay questions (25 points each), covering the core knowledge associated with the eight teaching cases. The examination was jointly developed by three senior experts who were not involved in teaching activities to ensure content validity. Cronbach’s α coefficient was 0.87.

##### 2.3.1.2 Case analysis ability assessment

After the intervention, students completed a standardized case analysis assessment (maximum score: 100 points) involving two novel clinical cases unrelated to the teaching sessions. Students were required to provide written responses within 60 min covering preliminary diagnosis, diagnostic basis, differential diagnosis, auxiliary examinations, treatment strategies, and prognostic evaluation. A structured scoring rubric (10–20 points per domain) was used. Two independent teachers scored the responses, and the average score was calculated. The intraclass correlation coefficient (ICC) was 0.91.

#### 2.3.2 Subjective recognition of the teaching model

##### 2.3.2.1 Teaching model evaluation questionnaire

Subjective recognition was captured using a 7-dimension “Teaching Model Evaluation Questionnaire,” adapted from established CBL scales and medical education satisfaction instruments to fit ophthalmology teaching characteristics. Items were constructed with reference to prior CBL studies and medical education quality literature, and content validity was confirmed by three senior ophthalmology educational experts. The seven dimensions assessed were: strong interactivity, immediate feedback, clinical relevance, enhanced knowledge understanding, cultivation of clinical thinking, promotion of self-directed inquiry, and provision of personalized learning experiences. Responses were scored using a three-level Likert scale (agree, basically agree, disagree).

##### 2.3.2.2 Overall teaching satisfaction

Overall teaching satisfaction was evaluated using a separate five-point Likert scale (very satisfied, satisfied, neutral, dissatisfied, and very dissatisfied). The overall satisfaction rate was calculated as follows:


$$\mathrm{overall}\,\mathrm{satisfaction}\,\mathrm{rate}=\frac{\mathrm{very}\,\mathrm{satisf}\,\text{ied}+\text{satisfied}}{\mathrm{total}\,\mathrm{number}\,\mathrm{of}\,\mathrm{participants}}$$

### 2.4 Statistical analysis

Statistical analyses were performed using SPSS version 27.0. Continuous variables are presented as mean ± standard deviation (SD). Between-group comparisons of continuous variables were conducted using independent-samples *t*-tests, while within-group pre- and post-intervention comparisons were performed using paired *t*-tests. Categorical variables are presented as frequencies and percentages and were analyzed using the chi-square (χ^2^) test.

To explore the pedagogical adaptability across different learner profiles, students were stratified based on their baseline comprehensive medical examination scores into high-foundation (≥ 80 points), medium-foundation (60–79 points), and low-foundation (< 60 points) subgroups. Inter-subgroup comparisons of score improvements and subjective parameters were performed using one-way analysis of variance (ANOVA).

For satisfaction scores derived from the five-point Likert scale, the Mann-Whitney U test was additionally applied to preserve ordinal information. For multiple comparisons involving the seven dimensions of the evaluation questionnaire, a Bonferroni correction was implemented, yielding an adjusted statistical significance threshold of *P* < 0.0071. For all other statistical tests, a two-sided *P* < 0.05 was considered statistically significant.

## 3 Results

### 3.1 Comparison of objective learning outcomes between groups

Before the intervention, no statistically significant difference was observed in theoretical examination scores between the experimental and control groups (64.5 ± 6.8 vs. 65.2 ± 7.1, *P* = 0.62). After the intervention, the experimental group achieved significantly higher theoretical examination scores than the control group (86.4 ± 5.2 vs. 78.9 ± 6.1, *t* = 6.51, *P* < 0.01). Similarly, case analysis assessment scores were significantly higher in the experimental group (84.7 ± 6.3 vs. 74.2 ± 7.5, *t* = 7.48, *P* < 0.01).

The average improvement in theoretical examination scores was 21.9 points in the experimental group compared with 13.7 points in the control group (*P* < 0.01).

### 3.2 Comparison of subjective recognition rates between groups

Recognition rates across all seven evaluation dimensions were significantly higher in the experimental group than in the control group (all *P* < 0.0071) (Table 2). The greatest difference was observed in the dimension of “personalized learning experience” (89.8% vs. 36.7%, χ^2^ = 32.15, *P* < 0.0071).

**TABLE 2:** Comparison of students’ recognition rates of teaching models [*n* (%)].

Evaluation item	Experimental group (*n* = 49)	Control group (*n* = 49)	χ ^2^	*P*
Strong interactivity	46 (93.9)	23 (46.9)	25.14	< 0.001
Immediate feedback	44 (89.8)	19 (38.8)	27.33	< 0.001
Clinical relevance	43 (87.8)	26 (53.1)	13.92	< 0.001
Enhanced knowledge understanding	42 (85.7)	24 (49.0)	14.92	< 0.001
Cultivation of clinical thinking	44 (89.8)	23 (46.9)	20.45	< 0.001
Promotion of self-directed inquiry	45 (91.8)	21 (42.9)	26.14	< 0.001
Personalized learning experience	44 (89.8)	18 (36.7)	32.15	< 0.001

### 3.3 Comparison of teaching satisfaction between groups

In the experimental group, the proportions of students reporting very satisfied, satisfied, neutral, dissatisfied, and very dissatisfied were 61.2% (30/49), 28.6% (14/49), 8.2% (4/49), 2.0% (1/49), and 0.0%, respectively, resulting in an overall satisfaction rate of 89.8%.

In the control group, the corresponding proportions were 22.4% (11/49), 40.8% (20/49), 24.5% (12/49), 10.2% (5/49), and 2.0% (1/49), respectively, with an overall satisfaction rate of 63.3%.

Analysis of the original ordinal satisfaction scores using the Mann-Whitney U test demonstrated that the experimental group possessed significantly higher teaching satisfaction than the control group (U = 652.5, *P* < 0.01).

### 3.4 Subgroup analysis according to academic foundation

After stratification according to comprehensive medical examination scores, the numbers of students and score improvements in each subgroup are presented in Table 3.

**TABLE 3:** Comparison of improvements in theoretical examination scores among students with different academic foundations (mean ± SD).

Academic foundation	Experimental group (*n* = 49)	Control group (*n* = 49)	Between-group difference	*P*-value
High	+18.2 ± 4.1 (*n* = 16)	+15.6 ± 3.9 (*n* = 15)	2.6	0.08
Medium	+22.3 ± 5.2 (*n* = 21)	+14.2 ± 4.5 (*n* = 22)	8.1	< 0.01
Low	+24.5 ± 5.8 (*n* = 12)	+10.1 ± 4.3 (*n* = 12)	14.4	< 0.01

Students with weaker academic foundations in the experimental group demonstrated significantly greater improvements in theoretical examination scores (+24.5 points) than those in the low-foundation subgroup of the control group (+10.1 points, *P* < 0.01). Their improvement was also significantly greater than that of students with strong academic foundations within the experimental group (+18.2 points, *P* < 0.05).

Furthermore, the recognition rate for “personalized learning experience” among low-foundation students in the experimental group (95.2%) was significantly higher than that among high-foundation students (81.2%, *P* < 0.05).

## 4 Discussion

This randomized parallel-controlled study demonstrated that integrating ChatGPT into a CBL-based ophthalmology teaching model significantly improved students’ theoretical knowledge acquisition, clinical case analysis abilities, and overall learning satisfaction compared with traditional lecture-based teaching. Notably, students with weaker academic foundations derived the greatest benefit from the intervention, demonstrating the efficacy of this innovative pedagogy in narrowing performance gaps.

The findings of this study are consistent with emerging evidence suggesting that AI-assisted educational tools may enhance learner engagement and promote self-directed learning in medical education. Unlike conventional lecture-based teaching, the CBL + ChatGPT model enabled students to actively participate in iterative clinical reasoning processes through interactive questioning and immediate feedback (36). From the perspective of constructivist learning theory, this approach may facilitate active knowledge construction by encouraging learners to continuously refine diagnostic reasoning through dynamic interactions, bridging the gap between theoretical knowledge and clinical application (37, 38).

Another important finding was the substantial improvement observed among students with weaker academic foundations. This may be explained by the personalized and low-pressure learning environment provided by ChatGPT. Because students could repeatedly query the AI system without concerns regarding embarrassment or teacher-imposed time limitations, individualized learning needs could be better addressed. This finding also aligns with principles of adaptive learning and educational equity, suggesting that AI-assisted teaching may serve as an effective equalizer, reducing disparities in learning efficiency among students with heterogeneous academic backgrounds (39).

Compared with previous studies, the present study possesses several methodological strengths. First, a randomized parallel-controlled design was adopted to minimize recall bias and social desirability bias commonly observed in retrospective educational studies. Second, objective outcome indicators, including standardized theoretical examinations and structured case analysis assessments, were incorporated in addition to subjective satisfaction evaluations. Third, a standardized human-AI interaction workflow combined with teacher supervision was established, thereby improving reproducibility and practical applicability.

Despite these strengths, several limitations should be acknowledged. First, the absence of participant and instructor blinding may have introduced potential implementation bias, although standardized teaching procedures were applied to minimize its impact. Second, although structured teacher supervision was implemented throughout the intervention, the possibility of inaccurate, incomplete, or hallucinated outputs generated by ChatGPT cannot be fully excluded (40, 41). Third, because the experimental design combined CBL with ChatGPT-assisted interactions and group discussions, the observed improvements may reflect the synergistic effect of these combined modalities rather than the contribution of ChatGPT in isolation. Fourth, the relatively small sample size within each academic foundation stratum may limit the statistical power of the subgroup analyses. Finally, the experimental group required additional real-time supervision of ChatGPT interactions, which may have introduced differences in instructor engagement or Hawthorne effects. These limitations highlight the need for cautious interpretation of the findings and underscore the importance of critical evaluation when integrating large language models into medical education.

Future multicenter randomized studies with extended follow-up periods are warranted to further evaluate the long-term effectiveness and educational sustainability of AI-assisted teaching models. Moreover, integrating educational theory frameworks, prompt engineering optimization, and clinical skill assessments may further improve the quality and standardization of AI-assisted medical education.

## 5 Conclusion

Compared with traditional multimedia lecture-based teaching, the CBL teaching model integrated with ChatGPT significantly improves theoretical knowledge acquisition and case analysis abilities in ophthalmology clinical education. It also achieves higher levels of student recognition and satisfaction, particularly among students with weaker academic foundations.

This teaching approach demonstrates strong potential for broader implementation and warrants further exploration and optimization in the context of digital transformation in medical education.

## Data Availability

The datasets presented in this article are not readily available because no datasets were generated or analyzed that are suitable for public sharing. Data supporting the findings are available from the corresponding author upon reasonable request and with permission of the ethics committee. Requests to access the datasets should be directed to Yuan Shen, sheny85@sjtu.edu.cn.

## References

[1] Rincón EHH Jimenez D Aguilar LAC Flórez JMP Tapia ÁER Peñuela CLJ. Mapping the use of artificial intelligence in medical education: a scoping review. *BMC Med Educ*. (2025) 25:526. 10.1186/s12909-025-07089-8 40221725 PMC11993958

[2] Ahsan Z. Integrating artificial intelligence into medical education: a narrative systematic review of current applications, challenges, and future directions. *BMC Med Educ*. (2025) 25:1187. 10.1186/s12909-025-07744-0 40849650 PMC12374307

[3] Zhao M Li J Li S Liu J Jiang Y Lai X et al. Artificial intelligence assisted simulation and surgical video analytics for ophthalmic surgery training and competence development. *Front Med (Lausanne)*. (2026) 13:1781818. 10.3389/fmed.2026.1781818 41939757 PMC13043345

[4] Gong D Li WT Li XM Wan C Zhou YJ Wang SJ et al. Development and research status of intelligent ophthalmology in China. *Int J Ophthalmol*. (2024) 17:2308–15. 10.18240/ijo.2024.12.20 39697896 PMC11589450

[5] Uzoechina G Osajiuba T. Use of large language models in undergraduate medical education a scoping review. *Disc Educ.* (2026) 5:266. 10.1007/s44217-026-01239-w

[6] Lucas HC Upperman JS Robinson JR. A systematic review of large language models and their implications in medical education. *Med Educ*. (2024) 58:1276–85. 10.1111/medu.15402 38639098

[7] Wei J Wang X Huang M Xu Y Yang W. Evaluating the performance of ChatGPT on board-style examination questions in ophthalmology: a meta-analysis. *J Med Syst*. (2025) 49:94. 10.1007/s10916-025-02227-7 40615678

[8] Maity S Saikia MJ. Large language models in healthcare and medical applications: a review. *Bioengineering (Basel)*. (2025) 12:631. 10.3390/bioengineering12060631 40564447 PMC12189880

[9] Ren X Fan C Ma W He H Gao C Zhao X et al. Medical reasoning with large language models: a systematic review and evaluation. *iNew Med.* (2026) e70056. 10.1002/inm3.70056 [Epub ahead of print].

[10] Jiang L Zhu Y Song C Hu X Fan X Yang W et al. Benchmarking large language models for congenital cataract parent counseling: safety, readability, and knowledge translation of developmental and genetic information. *Front Cell Dev Biol*. (2026) 14:1785731. 10.3389/fcell.2026.1785731 41970961 PMC13066122

[11] Cang X Ni M Song C Zhao J Guo Y Zou Y et al. ChatGPT-5 versus other mainstream large language models in core diabetic retinopathy patient queries. *Front Cell Dev Biol*. (2026) 14:1754221. 10.3389/fcell.2026.1754221 41960186 PMC13057549

[12] Xu X Chen Y Miao J. Opportunities, challenges, and future directions of large language models, including ChatGPT in medical education: a systematic scoping review. *J Educ Eval Health Prof*. (2024) 21:6. 10.3352/jeehp.2024.21.6 38486402 PMC11035906

[13] Zhui L Yhap N Liping L Zhengjie W Zhonghao X Xiaoshu Y et al. Impact of large language models on medical education and teaching adaptations. *JMIR Med Inform*. (2024) 12:e55933. 10.2196/55933 39087590 PMC11294775

[14] Jiang Y Jiang H Yang Z Li Y Chen Y. The application of novel techniques in ophthalmology education. *Front Med (Lausanne)*. (2024) 11:1459097. 10.3389/fmed.2024.1459097 39610687 PMC11602297

[15] Huang L Liu X Zou Y Lin X Mao Z. Integrated course on ocular anatomy for ophthalmology clerkship. *BMC Med Educ*. (2025) 25:3. 10.1186/s12909-024-06611-8 39748363 PMC11697863

[16] Lee R Raison N Lau WY Aydin A Dasgupta P Ahmed K et al. A systematic review of simulation-based training tools for technical and non-technical skills in ophthalmology. *Eye (Lond)*. (2020) 34:1737–59. 10.1038/s41433-020-0832-1 32203241 PMC7609318

[17] Wang X Liu B Wang M Zhang Z Zhu C Fu H et al. *Towards Clinically Interpretable Ophthalmic VQA via Spatially-Grounded Lesion Evidence.* arXiv:2605.22414 (2026).

[18] Tran JH Loebel E Edouard M Quehl T Walsh E Ginsburg R et al. Creating ophthalmology experiences in undergraduate medical education: pilot of a cased-based learning ophthalmology tool. *BMC Med Educ*. (2023) 23:559. 10.1186/s12909-023-04514-8 37559068 PMC10410917

[19] Krungkraipetch L Krungkraipetch N Leelawongs S. Global disparities in ophthalmology education and alignment with international council of ophthalmology guidelines among medical students: a systematic review and meta-analysis. *BMC Med Educ*. (2025) 25:452. 10.1186/s12909-025-07012-1 40148915 PMC11951766

[20] Zhang SL Ren SJ Zhu DM Liu TY Wang L Zhao JH et al. Which novel teaching strategy is most recommended in medical education? A systematic review and network meta-analysis. *BMC Med Educ*. (2024) 24:1342. 10.1186/s12909-024-06291-4 39574112 PMC11583476

[21] Zhang W Wei J Guo W Wang Z Chen S. Comparing the effects of team-based and problem-based learning strategies in medical education: a systematic review. *BMC Med Educ*. (2024) 24:172. 10.1186/s12909-024-05107-9 38388937 PMC10885648

[22] Xiong X Xu J Luo M Niu D Bi Q Wang Z et al. Efficacy of problem-based learning combined with case-based learning versus lecture-based learning in orthopedic education: a systematic review and meta-analysis. *BMC Med Educ*. (2025) 25:1357. 10.1186/s12909-025-07741-3 41053695 PMC12502553

[23] Daly R Tunney E Spooner M Offiah G Flood K Kent F. Case-based learning (CBL) in undergraduate health professions education: a realist review. *Med Educ.* (2026) 1–19. 10.1111/medu.70179 [Epub ahead of print]. 41638636 PMC13350282

[24] Maia D Andrade R Afonso J Costa P Valente C Espregueira-Mendes J. Academic performance and perceptions of undergraduate medical students in case-based learning compared to other teaching strategies: a systematic review with meta-analysis. *Educ Sci.* (2023) 13:238. 10.3390/educsci13030238

[25] Tegginamani AS Vanishree HS. The benefits and limits of case-based learning (CBL): a concise review. *J Multidisc Dental Res.* (2024) 10:74–80. 10.38138/JMDR/v10i2.26

[26] Kung TH Cheatham M Medenilla A Sillos C De Leon L Elepaño C et al. Performance of ChatGPT on USMLE: potential for AI-assisted medical education using large language models. *PLoS Digit Health*. (2023) 2:e0000198. 10.1371/journal.pdig.0000198 36812645 PMC9931230

[27] Safarov R Wippermann J Meyer F Wacker M. Problem-based learning in the age of generative AI: a structured blueprint for medical curricula. *Z Evid Fortbild Qual Gesundhwes*. (2026) 203:87–96. 10.1016/j.zefq.2026.03.012 42225450

[28] Zeng J Sun K Qin P Liu S. Enhancing ophthalmology students’ awareness of retinitis pigmentosa: assessing the efficacy of ChatGPT in AI-assisted teaching of rare diseases-a quasi-experimental study. *Front Med (Lausanne)*. (2025) 12:1534294. 10.3389/fmed.2025.1534294 40171502 PMC11959056

[29] Wulandari ANE Purwono P Ma’arif A Marquez Vera MA Majdoubi R Salah WA. ChatGPT as a pedagogical tool for clinical reasoning in medical education: a systematic narrative review. *J Technol Pedagogy Educ Dev.* (2025) 2:12–28.

[30] Abidi SH Almazan J Fabiyi O Zehra F Tariq M. AI-supported case-based learning in medical education: a comprehensive scoping review. *Front Med (Lausanne)*. (2026) 13:1798097. 10.3389/fmed.2026.1798097 41958555 PMC13056847

[31] Han JW Park J Lee H. Analysis of the effect of an artificial intelligence chatbot educational program on non-face-to-face classes: a quasi-experimental study. *BMC Med Educ*. (2022) 22:830. 10.1186/s12909-022-03898-3 36457086 PMC9713176

[32] Ouyang F Wu M Zheng L Zhang L Jiao P. Integration of artificial intelligence performance prediction and learning analytics to improve student learning in online engineering course. *Int J Educ Technol High Educ*. (2023) 20:4. 10.1186/s41239-022-00372-4 36683653 PMC9842403

[33] Xu W Ouyang F. A systematic review of AI role in the educational system based on a proposed conceptual framework. *Educ Informat Technol.* (2022) 27:4195–223. 10.1007/s10639-021-10774-y

[34] Mallik S Gangopadhyay A. Proactive and reactive engagement of artificial intelligence methods for education: a review. *Front Artif Intell*. (2023) 6:1151391. 10.3389/frai.2023.1151391 37215064 PMC10196470

[35] Antaki F Touma S Milad D El-Khoury J Duval R. Evaluating the performance of ChatGPT in ophthalmology: an analysis of its successes and shortcomings. *Ophthalmol Sci*. (2023) 3:100324. 10.1016/j.xops.2023.100324 37334036 PMC10272508

[36] Abidi SH Almazan J Zehra F Fabiyi O Tariq M. Role of AI-supported case-based learning in medical education: a scoping review protocol. *BMJ Open*. (2025) 15:e109397. 10.1136/bmjopen-2025-109397 41469057 PMC12750761

[37] Holmes W Bialik M Fadel C. *Artificial Intelligence in Education. Promise and Implications for Teaching and Learning.* Boston (MA): Center for Curriculum Redesign (2019).

[38] Zawacki-Richter O Marín VI Bond M Gouverneur F. Systematic review of research on artificial intelligence applications in higher education – where are the educators? *Int J Educ Technol High Educ.* (2019) 16:39. 10.1186/s41239-019-0171-0

[39] Luckin R Holmes W. *Intelligence Unleashed: An Argument for AI in Education.* London (UK): Pearson (2016).

[40] Farquhar S Kossen J Kuhn L Gal Y. Detecting hallucinations in large language models using semantic entropy. *Nature*. (2024) 630:625–30. 10.1038/s41586-024-07421-0 38898292 PMC11186750

[41] Huang L Yu W Ma W Zhong W Feng Z Wang H et al. A survey on hallucination in large language models: principles, taxonomy, challenges, and open questions. *ACM Trans Inf Syst.* (2025) 43:42. 10.1145/3703155

